# Potential Renoprotective Agents through Inhibiting CTGF/CCN2 in Diabetic Nephropathy

**DOI:** 10.1155/2015/962383

**Published:** 2015-09-02

**Authors:** Songyan Wang, Bing Li, Chunguang Li, Wenpeng Cui, Lining Miao

**Affiliations:** ^1^Department of Nephrology, Second Hospital of Jilin University, Changchun 130041, China; ^2^Department of Nephrology, Jilin Province People's Hospital, Changchun 130021, China; ^3^Department of Urology, The 2nd Hospital of Changchun, Changchun 130061, China

## Abstract

Diabetic nephropathy (DN) is the leading cause of end-stage renal disease (ESRD). The development and progression of DN might involve multiple factors. Connective tissue growth factor (CCN2, originally known as CTGF) is the one which plays a pivotal role. Therefore, increasing attention is being paid to CCN2 as a potential therapeutic target for DN. Up to date, there are also many drugs or agents which have been shown for their protective effects against DN via different mechanisms. In this review, we only focus on the potential renoprotective therapeutic agents which can specifically abolish CCN2 expression or nonspecifically inhibit CCN2 expression for retarding the development and progression of DN.

## 1. Introduction

Diabetes is one of the manifestations of metabolic syndrome, characterized by high blood glucose. Diabetic nephropathy (DN), as one of the most severe chronic diabetic microvascular complications, is a leading cause of end-stage renal disease (ESRD), which may result in high morbidity and mortality [1]. The pathological features of DN include glomerular and tubuloepithelial hypertrophy, diffuse thickening of glomerular and tubular basement membranes, mesangial expansion, and extracellular matrix proteins accumulation in the mesangium and tubulointerstitium, which may finally lead to glomerulosclerosis and tubulointerstitial fibrosis. Many factors and molecules contribute to this pathophysiological process, such as chronic hyperglycemia (HG), transforming growth factor-*β*1 (TGF-*β*1), and advanced glycation end product (AGE) [2]. Among them, CCN2 appears to play an important role in the development of DN.

Connective tissue growth factor (CTGF/CCN2) is a cytokine which was firstly detected by Bradham from conditioned human umbilical vein endothelial cells in 1991 [3]. It is a 38 kD cysteine-rich peptide that belongs to the emerging CCN (CYR61, CTGF, NOV) family of multifunctional growth factors [4]. CCN2 is widely expressed in human tissues and organs, such as adult connective tissue, heart, brain, kidney, lung, liver, muscle, pancreas gland, and placenta, especially high in the kidney [5]. CCN2 was found in glomerular cells, tubular epithelial cells, and interstitial cells of the diabetic kidneys [6, 7]. And CCN2 was upregulated in glomeruli of streptozotocin- (STZ-) induced diabetic rats with nephropathy and in primary human mesangial cells stimulated by glucose [8]. TGF-*β*1, AGE, and angiotensin II (Ang II) also induced CCN2 expression under diabetic conditions [9–11]. The overproduction of CCN2 is suggested to play a pivotal role in some fibrotic diseases, including renal fibrosis [4].

Up to date, accumulating evidence suggests that CCN2 is not only a marker but also a key mediator in DN [12]. CCN2 contributes to the progress of DN through its function. CCN2 prevented matrix degradation through increasing the tissue inhibitor of matrix metalloproteinases 1 (TIMP-1) expression in diabetes [13]. CCN2 also caused epithelial to mesenchymal transition (EMT) in renal tubular cells in diabetes, leading to genesis of new fibroblasts in the renal interstitium [14, 15]. Moreover, CCN2 promoted kidney fibroblast proliferation and ECM synthesis [14, 16]. In vivo study showed that overexpression of CCN2 in podocytes worsened proteinuria and mesangial expansion through a functional impairment and loss of podocytes in mice with DN [17]. CCN-2 also contributed to the renal podocyte apoptosis [18]. In addition, CCN-2 induced proinflammatory cytokines and led to early renal inflammation that preceded overt DN [19]. Remarkably the level of CCN2 expression was shown to be related with the severity and progression of renal fibrosis [20, 21] and it was proposed to be a useful molecular marker for the fibrotic response [22].

In recent years, with deeply understanding the role of CCN2 in fibrotic diseases including DN, CCN2 has become an important molecular marker in DN. And CCN2 has also become an index to auxiliarily verify the protective effect of some drugs or agents on diabetic nephropathy. In this review, we will summarize some potential renoprotective agents which can wholly or partly inhibit CCN2 for delaying the development and progression of diabetic kidney disease.

## 2. Agents Which Can Specifically Inhibit CCN2 Expression in DN

Based on the profibrotic role of CCN2 in diabetic nephropathy kidney disease, specific inhibitors of CCN2 such as CCN2 antisense oligonucleotides and neutralizing antibodies to CCN2 (FG-3019) have been studied (Table 1).

### 2.1. CCN2 Antisense Oligonucleotide (ASO)

Previous studies demonstrated that blockade of CCN2 by antisense oligodeoxynucleotide (ODN) suppressed the production of fibronectin and type I collagen in renal fibroblasts exposed to TGF-*β*1 and attenuated renal tubulointerstitial fibrosis [23]. Intravenous administration of CCN2 antisense ODN to subtotally nephrectomized TGF-*β*1 transgenic mice led to marked reduction of CCN2 expression in the proximal tubular epithelial cells in the remnant kidney, accompanied by the decrease of matrix molecules, plasminogen activator inhibitor-1 (PAI-1), and tissue inhibitor of metalloproteinase-1, and with no change of TGF-*β*1 levels [24]. CCN2 ASO also significantly decreased the expression of CCN2, fibronectin, fibronectin ED-A, and alpha1 (I) collagen genes in obstructed kidneys and without affection of TGF-*β* gene upregulation in unilateral ureteral obstruction (UUO) rats [25]. These findings indicate the important role of the TGF-*β*1-independent CCN2 pathway in the development of renal interstitial fibrosis.

In addition, CCN2 ASO significantly attenuated angiotensin II- (Ang II-) induced epithelial mesenchymal transition (EMT), as evidenced by markedly decreased alpha-SMA expression in human proximal tubular cell line (HK2) [26]. Guha et al. also evaluated the role of the specific blockade of CCN2 with a CCN2 ASO of novel chimeric chemistry (phosphorothioate and phosphodiester) in the progression of DN with a convenient biweekly dose schedule. In the streptozotocin- (STZ-) induced type 1 DN model of mice, hyperglycemic animals treated with CCN2 ASO (20 mg/kg/2 qw) for 4 months showed dramatically reduced CTGF expression in the kidney and decreased proteinuria and albuminuria. In type 2 DN model of db/db mice, administration of the CCN2 ASO for 8 weeks not only reduced serum creatinine and attenuated urinary albuminuria and proteinuria, but also decreased the expression of fibronectin, collagen (I and IV), and PAI-1 in the renal cortex through inhibiting phosphorylation of p38 MAPK and its downstream target CREB pathway. In vitro study showed that CCN2 ASO suppressed CCN2 and ECM protein expression in rat mesangial cells (MCs) exposed to high glucose [27]. These results suggest that specific blockade of CCN2 by a chimeric ASO is very promising in delaying the development and progress of diabetic nephropathy.

### 2.2. CCN2 Monoclonal Antibody-FG-3019

A human monoclonal antibody to CCN2, FG-3019, was initially used in treating a variety of tumors experimentally and showed antitumor activities in pancreas cancer [28], metastatic melanoma [29], and B-acute lymphoblastic leukemia (ALL) [30]. In addition, FG-3019 attenuated left ventricular remodeling and left ventricular dysfunction in pressure overload-induced heart failure [31]. FG-3019 was also used in the experimental treatment of diabetic nephropathy. In a recent phase I clinical study, a human monoclonal antibody to CCN2, FG-3019, was intravenously administered to patients with microalbuminuric diabetic kidney disease (DKD) (*n* = 24) with the dose of 3 or 10 mg/kg every 2 weeks for four doses and then followed up at days 62 and 365. The trial results showed that urinary albumin/creatinine ratio (ACR) reduced dramatically from 48 mg/g ACR (at baseline) to 20 mg/g ACR (day 56) (*P* = 0.027). There seems to be mild infusion adverse events on infusion day, but no significant drug-related side effect was observed over one year of follow-up [32]. Although reduction of albuminuria by FG-3019 in DKD patients was promising, the efficacy need to be further validated in a prospective, randomized, blinded study. Taken together, CTGF monoclonal antibody may become a potential therapeutic agent for diabetic kidney disease.

## 3. Other Renoprotective Agents Which Can Nonspecifically Inhibit CCN2 Expression in DN

Up to date, a wide variety of agents or drugs have shown their renoprotective properties through different mechanisms in diabetic nephropathy, but not all of the agents have the ability to inhibit the expression of connective tissue growth factor (CCN2). CCN2, as an important profibrotic cytokine, contributes to the development and progression of DN. Therefore, in spite of the specific CCN2 inhibitors (CCN2 ASO and CCN2 monoclonal antibody-FG-3019), we also address several related agents which hold potential renoprotective effects against DN at least partly through inhibiting CCN2 expression. Some of the pathways between these renoprotective agents and CCN2 expression have been elucidated, but still several unknown related pathways/mechanisms need to be further studied. These agents include renin-angiotensin- aldosterone system (RAAS) inhibitors, Rho Kinase Inhibitors, statins, mycophenolate mofetil, pyridone agents, glucagon-like peptide-1 (GLP-1) analog, and purple corn anthocyanins (PCA) (Table 2).

### 3.1. (Pro)Renin Receptor (PRR) Blockade

**Figure 1:**
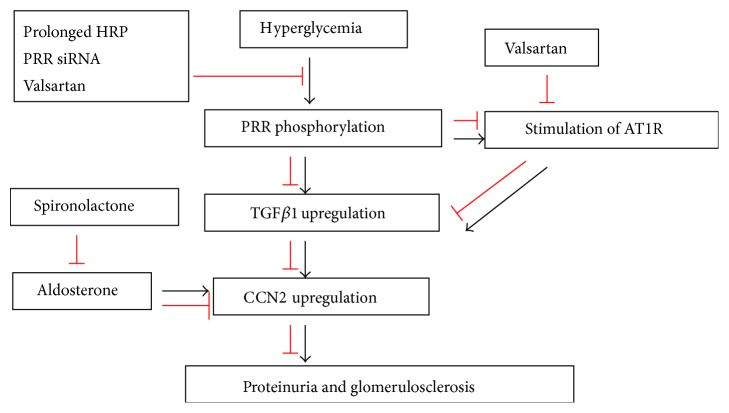
Effect of renin-angiotensin-aldosterone system (RAAS) inhibitors on CCN2 expression. Rapid (Pro)renin receptor (PRR) phosphorylation induced by high glucose was prior to upregulation of TGF*β*1 and CCN2, and PRR phosphorylation was inhibited by prolonged handle region peptide (HRP) of prorenin, valsartan, and PRR siRNA treatments, which suggested that TGF*β*-CCN2 axis was activated by PRR signaling pathway. Combined treatment with valsartan and PRR siRNA further decreased TGF*β*1 and CCN2 expression, suggesting that AT1R and PRR may independently influence TGF*β*1-CCN2 axis, or PRR upregulated TGF*β*1 and CCN2 expression by promoting angiotensin II formation and stimulation of AT1R [30]. Aldosterone directly induced CCN2 overproduction through a TGF-beta1-independent pathway. Spironolactone markedly suppressed the production of CCN2 induced by aldosterone [46].

PRR is a new member of RAS. PRR acts by binding and activating prorenin through a single transmembrane domain [33]. It was reported that PRR was upregulated in kidneys of diabetic rats and renal mesangial cells (RMCs) induced by high glucose [34, 35]. A recent study has shown that (Pro)renin receptor (PRR) promoted the progression of diabetic nephropathy through enhanced TGF*β*1 CCN2 signaling cascade, which was evidenced by administering PRR blockade in vivo and in vitro. High glucose-induced rapid PRR phosphorylation (30 min) was prior to upregulation of TGF*β*1 and CCN2, and PRR phosphorylation was inhibited by prolonged handle region peptide (HRP) of prorenin, valsartan, and PRR siRNA treatments, which suggested that TGF*β* CCN2 axis was activated by PRR signaling pathway. PRR blockade markedly decreased TGF-*β*1 and CCN2 expression in diabetic animals and high glucose treated rat mesangial cells (RMCs). Angiotensin AT1 receptor blockade with valsartan got similar effects. Combined treatment with valsartan and PRR siRNA further decreased TGF*β*1 and CCN2 expression, suggesting that AT1R and PRR may independently influence TGF*β*1 CCN2 axis, or PRR upregulated TGF*β*1 and CCN2 expression by promoting angiotensin II formation and stimulation of AT1R (Figure 1) [36]. Other studies also demonstrated that PRR blockade attenuated albuminuria in diabetic rats [37] and delayed the progression of diabetic nephropathy [38]. Taken together, the blockade of AT1R and PRR may be renoprotective through inhibiting TGF*β*1-CCN2 expression.

### 3.2. Angiotensin II Receptor Blockade

It was reported that the intrarenal renin-angiotensin system (RAS) was aberrantly activated in the prediabetic stage and might promote the onset and development of later diabetic nephropathy [39]. Previous studies have reported that angiotensin II (Ang II) contributed to renal hypertrophy and subsequent renal fibrosis in the development of diabetic nephropathy [40]. CCN2 is also involved in renal hypertrophy induced by angiotensin (Ang II). It was reported that the expression of CCN2 was markedly upregulated in both glomeruli and tubuli of diabetic rats, and the extent of CCN2 expression closely correlated with the severity of renal hypertrophy, administration of irbesartan (IRB), or Ang II receptor antagonist significantly suppressed CCN2 expression in kidneys of diabetic rats [41, 42]. The in vitro study showed that angiotensin II (Ang II) significantly stimulated CCN2 expression in human proximal tubular cells (HK2 cells). Moreover, Ang II promoted the increase of HK2 cell size and arrested the cell cycle in the G0-G1 phase, which was notably reversed by cotreatment with CTGF ASO [41]. In addition, a three-year clinical study, including 71 hypertensive type 1 diabetic nephropathy patients who were treated with Losartan, demonstrated that Losartan markedly decreased urinary CCN2 by 21% initially (*P* < 0.05 versus baseline), with no further attenuation after increasing dose. The continuous reduction in urinary CTGF was 22% (*P* < 0.05 versus baseline). The persistent reduction of the urinary CCN2 excretion by Losartan correlated with a slower rate of decline in GFR, in spite of plasma CCN2 remaining unchanged throughout the study [43]. These data indicate that the relationship of angiotensin II receptor blockade and CCN2 expression and angiotensin II receptor blockade exerts its renoprotective effect partly through reduction of CTGF expression.

### 3.3. Aldosterone Receptor Blockade—Spironolactone

Aldosterone is regarded as an injurious component of the renin-angiotensin-aldosterone system in renal tissue [44]. Aldosterone receptor blockade also provides beneficial effects in patients with early type 2 diabetic nephropathy [45]. The direct relationship of aldosterone and CCN2 expression in diabetic nephropathy had also been studied. And the results showed that aldosterone upregulated the expression of CCN2, type I and type IV collagen production, in a dose-dependent manner in cultured mesangial cells (MCs) and proximal tubular cells (PTCs), without affection of TGF-*β*1 gene expression and protein synthesis. Blockade of aldosterone with spironolactone, a nonspecific mineralocorticoid receptor (MR) antagonist, markedly suppressed the production of CCN2 and collagen induced by aldosterone. But inhibition of TGF-*β*1 with neutralizing TGF-*β*1 antibody did not influence aldosterone-induced CCN2 synthesis, which indicated that aldosterone might directly induce CCN2 overproduction through a TGF-*β*1-independent pathway in cultured MCs and PTCs. Moreover, spironolactone treatment reduced urinary protein and albumin excretion and prohibited the glomerulosclerosis which correlated with reduction of CCN2, type I and type IV collagen expression in type 2 diabetes mellitus rat model (Figure 1) [46]. Furthermore, recent clinical trials showed that spironolactone alone effectively decreased proteinuria in microalbuminuric patients with type 1 or type II diabetes [47–49], which was independent of blood pressure [47]. And combination of spironolactone (50 mg/day) with hydrochlorothiazide (25 mg/day) significantly decreased 24 h urine protein and did not increase serum potassium in type 2 diabetic patients with microalbuminuria [48]. These findings suggest that spironolactone may provide the renal protective role at least partly through downregulating the expression of CCN2 in early diabetic nephropathy.

### 3.4. Rho Kinase Inhibitors

Recent studies also have shown that the small GTPase Rho and its downstream effector Rho-associated kinases (ROCKs) played a crucial role in renal disease including diabetic nephropathy, and selective ROCK inhibitors, such as fasudil and Y-27632, attenuated the development of diabetic nephropathy. It was reported that Rho/Rho-kinase pathway correlated with the increase of transforming growth factor-beta (TGF-*β*) and connective tissue growth factor (CCN2) in diabetes, and the RhoA/Rho-kinase was activated in diabetic db/db mice and rat mesangial cells (MCs) exposed to high glucose [50, 51]. Early administration of selective ROCK inhibitor fasudil significantly suppressed the expression of TGF-*β* and CCN2 in the renal cortex, attenuated glomerulosclerosis and renal interstitial fibrosis, and decreased urinary albumin excretion [52] without affection on blood glucose or blood pressure in diabetic rats [53]. The reduction of TGF-*β* and CCN2 expression in renal cortex of diabetic rats caused by fasudil suggested that the Rho/Rho kinase pathway was involved in the upregulation of TGF-*β* and CCN2 in diabetic kidney. The in vitro study also demonstrated that fasudil reduced CCN2, FN, and TNF*α* protein secretion via suppression the activation of Rho/ROCK signaling pathway and attenuated the inflammation and fibrosis of high glucose-activated HMCs [54]. Taken together, these accumulated findings strongly indicate that RhoA/Rho inhibitors are potential therapeutic agents in the treatment of diabetic renal disease by different mechanisms including inhibition of CCN2.

### 3.5. Statins

Statins, inhibitor of 3-hydroxy-3-methylglutaryl CoA reductase, are widely administered to control blood cholesterol levels clinically. Some studies have demonstrated that statins played renoprotective roles in several glomerular diseases, including DN [55, 56]. Moreover, administration of simvastatin with the dose of less than cholesterol-lowering (2 mg/kg/d) attenuated the progression of the tubulointerstitial fibrosis by reducing the expression of CCN2 and *α*-SMA in renal tubulointerstitium of diabetic nephropathy rats [57]. Furthermore, simvastatin inhibited CCN2 secretion induced by high glucose in cultured human mesangial cells and prohibited HG induced transcription of the CCN2 promoter in transfected HEK293 cells significantly; the possible mechanism of CCN2 transcription inhibition with simvastatin might be due to the inhibition of NF-*κ*B activation pathways [58, 59] or impaired glucose uptake and decreased the intracellular concentrations of D-glucose [60]. But the inhibitory effect of statins on urine and plasma CCN2 was not observed in 405 subjects with/without type 2 diabetes; the putative reasons might be several associated factors involved in the expression of CCN2 [61]. Additionally, simvastatin also significantly decreased albuminuria and mesangial matrix expansion in the kidney cortices of db/db mice [50]. Overall, statins might be a renoprotective drug partly via the inhibition of CCN2 expression.

### 3.6. Mycophenolate Mofetil

Mycophenolate mofetil (mycophenolate mofetil, MMF) is a new, efficient immunosuppressor. MMF plays its role of immunosuppression mainly by noncompetitive and reversible inhibition of the rate-limiting enzyme-inosine monophosphate dehydrogenase (IMPDH) for de novo purine synthesis to strongly inhibit T, B lymphocyte proliferation. Thus MMF is more widely used in the treatment of organ transplantation, bone marrow transplantation, and autoimmune diseases. Recent studies have shown that MMF can also be used in treating diabetic nephropathy. MMF reduced protein urine in STZ-induced type-1 diabetic nephropathy model [62]. MMF also decreased the expression of CCN2 in mesangial cells (MCs) exposed to high glucose. The possible mechanism of MPA reduction of CCN2 expression might be that MPA could interfere with G protein-regulated intracellular signal pathway. G protein is activated when GTP is the binding nucleotide; then the downstream signal pathway is switched on. When the binding nucleotide is GDP, G protein is biodeactivated, and the downstream signal pathway is shut down. MPA might downregulate the expression of TGF-*β* and CCN2 by reducing intracellular GTP accumulation via suppressing the activity of IMPDH, deactivating most G protein and blocking intracellular signal pathway [63]. A recent study also demonstrated that the expression of monocyte chemoattractant protein-1 (MCP-1) as well as the secretion of fibronectin (FN) was inhibited by MMF in human mesangial cells (HMCs) exposed to high glucose [64]. These results suggested that MMF played the antifibrotic effect on diabetic nephropathy through suppressing CCN2 and FN expression.

### 3.7. Pyridone Agents

Pyridone agents have antifibrotic properties. Fluorofenidone [1-(3-fluorophenyl)-5-methyl-2-(1H)-pyridone, AKF-PD], a novel pyridone agent, has shown potent antifibrotic capabilities. AKF-PD significantly reduced TGF-*β*1-induced CCN2 expression in mouse mesangial cells (MMCs). Administration of PD98059 (Erk inhibitor) and SB203580 (P38 inhibitor), respectively, significantly downregulated TGF-*β*1-induced CCN2 expression in mouse mesangial cells (MMCs), suggesting that the downregulation of fluorofenidone on CCN2 expression induced by TGF-*β*1 might be through ERK and p38 pathways [65]. Moreover, fluorofenidone (AKF-PD) also markedly suppressed TGF-*β*1-induced tubular epithelial-mesenchymal transition (EMT) and connective tissue growth factor (CCN2) expression in human proximal tubular epithelial cells (HK2) through blocking TGF-*β*/Smads signaling [66]. In vivo study also showed that fluorofenidone attenuated renal interstitial fibrosis in the rat model of obstructive nephropathy caused by unilateral ureteral obstruction (UUO) through its reduction of the expression of *α*-SMA, TGF-*β*1, CCN2, platelet-derived growth factor (PDGF), and inhibitor of TIMP-1 in the obstructed kidneys [67]. AKF-PD also reduced renal fibrosis and renal dysfunction through decreasing the abnormal accumulation of mesangial matrix by suppressing upregulated expression of TGF-*β* target genes in kidneys of db/db mice [68]. This accumulated evidence suggests that pyridone agents are novel treatment approaches by reducing the rate of renal function decline for diabetic nephropathy.

### 3.8. Glucagon-Like Peptide-1 (GLP-1) Analog—Exendin-4

Exendin-4 is an analog of glucagon-like peptide-1 (GLP-1), which is a gut incretin hormone and is considered a potential therapeutic drug for type 2 diabetes. Exendin-4 was firstly isolated from the salivary secretions of the Gila monster lizard [69]. It exerts its glucose-controlling effect by binding to and activating GLP-1 receptor [70], stimulating insulin secretion, inhibiting glucagon secretion, inducing satiety, and delaying gastric emptying [71, 72]. Exendin-4 also exerted potential protective role in diabetic nephropathy by prohibiting high glucose-induced human mesangial cells (HMCs) proliferation and decreasing the expression of TGF-*β*1 and CCN2. The intracellular signaling cascade of CCN2 reduction by Exendin-4 was also investigated by preincubating HMCs with MDL-12330A, a specific adenylyl cyclase inhibitor, and PKI14-22, a specific protein kinase A (PKA) inhibitor, and the results showed that MDL-12330A and PKI14-22 markedly reversed the inhibitory effect of Exendin-4 on TGF-*β*1 and CCN2 mRNA, respectively, suggesting the inhibitory effect of Ex-4 on TGF-*β*1 mRNA and CCN2 mRNA partly via the cAMP/PKA pathway [73]. The in vivo study demonstrated that intraperitoneal administration of Exendin-4 (1 nmol/kg/day) to db/db mice for 8 weeks significantly attenuated glomerular hypertrophy, mesangial matrix expansion, TGF-*β*1 expression, type IV collagen accumulation, and associated glomerular lipid accumulation, together with fewer infiltrating inflammatory cells and apoptotic cells in the glomeruli, which indicated the therapeutic role of Exendin-4 in type 2 diabetic nephropathy [74]. Although several animal studies reported that GLP-1 receptor agonists have protective roles in diabetic nephropathy independent of their glucose-lowering effect, another report elucidated that exenatide should not be administered in patients with severe renal impairment or end stage renal disease [75]. Therefore, further research is needed to investigate the role of glucagon-like peptide-1 (GLP-1) anolog in diabetic nephropathy.

### 3.9. Purple Corn Anthocyanins (PCA)

**Figure 2:**
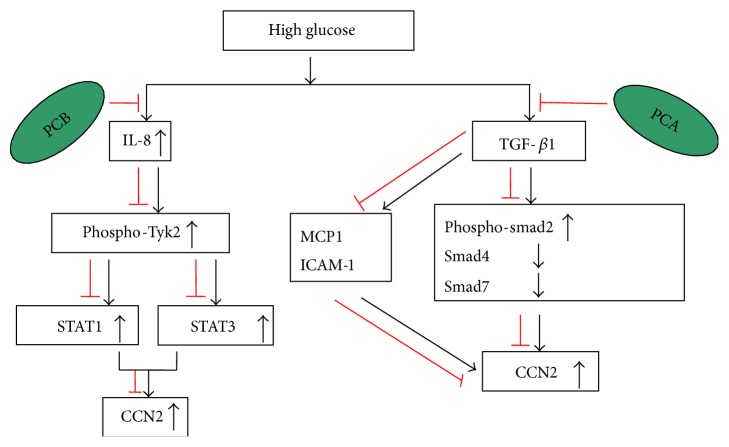
Effect of purple corn anthocyanins (PCA) on CCN2 expression. Anthocyanin-rich purple corn butanol fraction (PCB) concentration dependently mitigated HG-inflamed induction of CCN2. PCB also greatly inhibited HG-induced IL-8 secretion and the activation of Tyk2 and STAT1 and STAT3 in mesangial cells. Therefore, HG-provoked glomerular injury of fibrosis caused by mesangial inflammation of IL-8 was activated by Tyk2-STAT signaling pathway [75]. In addition, HG-stimulated CCN2 induction in human mesangial renal cells (HRMC) correlated with TGF-*β*-SMAD-responsive pathways which were blunted by PCA, evidenced by dampening HG-promoted SMAD2 phosphorylation and SMAD4 expression and reversing HG-inhibited SMAD7 expression in HRMCs. Moreover, PCA attenuated TGF-*β*-triggered inflammatory ICAM-1 expression and MCP-1 production in the mesangium, leading to the reduction of CCN2 expression [79].

Purple corn, rich in anthocyanins, has been considered as a functional food and holds potential disease-preventive properties in diabetes and diabetic complications. It was reported that dietary purple corn color (PCC) attenuated high fat (HF) diet-induced insulin resistance in mice [76]. In addition, purple corn anthocyanins (PCA) not only ameliorated blood glucose level and HbA1c, but also protected beta cell from cell death in HIT-T15 cell culture and db/db mice [77]. Moreover, recent studies have shown that PCA weakened the fibrosis at least partly via downregulating CCN2 expression in human renal mesangial cells treated with high glucose and db/db mice. High glucose promoted cellular expression and secretion of CCN2 in human renal mesangial cells (HRMCs). IL-8 is involved in this process. High glucose augmented the production of the chemokine IL-8 in HRMC, leading to Tyk2 phosphorylation and the activation of STAT1 and STAT3 (Tyk2 downstream proteins). However anthocyanin-rich purple corn butanol fraction (PCB) concentration dependently mitigated HG-inflamed the induction of CCN2 and collagen IV secretion. In addition, PCB greatly inhibited high glucose-induced IL-8 secretion and the activation of Tyk2 and STAT1 and STAT3 in mesangial cells. Therefore, HG-provoked glomerular injury of fibrosis caused by mesangial inflammation of IL-8 was activated by Tyk2-STAT signaling pathway. Moreover, the in vivo study revealed that the anthocyanin-rich polyphenolic extracts of purple corn (PCE) supplement to db/db mice significantly extenuated the expression of TGF-beta, CCN2, and collagen IV in db/db mouse renal tissues. PCE also attenuated plasma glucose level and severe albuminuria. Furthermore, the expressions of nephrin and podocin were inhibited by treating mice with PCE [78]. Another study also revealed that the HG-stimulated CCN2 induction in HRMC correlated with TGF-*β*-SMAD-responsive pathway which was blunted by PCA. PCA dampened HG-promoted SMAD2 phosphorylation and SMAD4 expression and also reversed HG-inhibited SMAD7 expression in HRMCs. In addition, PCA alleviated HG-inflamed hyperplasia and CCN2-induced ECM expansion through improving matrix degrading MMP system involving TIMP-2. Moreover, PCA attenuated TGF-*β*-triggered inflammatory intercellular cell adhesion molecule-1 (ICAM-1) expression and MCP-1 production in the mesangium, leading to the reduction of CCN2 expression (Figure 2). Furthermore, PCA disturbed the crosstalk between TGF-*β* and NF-*κ*B signaling which mediated diabetes-associated mesangial inflammation-linked renal fibrosis [79]. Due to the capability of PCA in lessening mesangial inflammation-associated renal fibrosis, PCA seems to be very promising in retarding the progression to diabetic nephropathy.

### 3.10. Aminoguanidine (AG)

Aminoguanidine (AG), a nonspecific inhibitor of advanced glycation end products (AGEs) formation, plays its role by scavenging intermediates in the advanced glycation catalytic process. In the development of DN, AGEs are accumulated and promote the pathological process [80]. Both soluble and matrix bound AGEs upregulated CCN2 expression in cultured human renal mesangial cells [81, 82]. AGEs also increased the expression of CCN2 in tubular epithelial cells (TECs) [10]. Based on the role of AGEs in CCN2 induction in DN, such anti-AGEs agent as AG has been studied and certified the effect of CCN2 inhibition in experimental diabetic models. It was reported that treatment of diabetic rats with AG prevented the induction of CCN2 at the level of mRNA and protein in the kidneys by suppressing AGEs formation. AG also prevented both AGE and CCN2 immunostaining in the renal cortices of diabetic rats. These data suggest that AG might prevent glomerular damage in diabetes via inhibition of CCN2 [81]. To test whether AG, also known as pimagedine, could attenuate nephropathy in type 1 diabetes mellitus, a randomized, double-masked, placebo-controlled clinical study was performed in 690 patients with type 1 diabetes mellitus, nephropathy, and retinopathy. The final results showed that pimagedine reduced the 24-hour total urinary proteinuria (*P* ≤ 0.001) even though the primary end point was not achieved [83]. But it was also evidenced that aminoguanidine caused DNA damage by free radical production [84]. Therefore, the administration of AG to diabetic nephropathy is restricted considering the safety.

### 3.11. Tranilast

Tranilast (N-[3,4-dimethoxycinnamoyl]anthranilic acid), a synthetic inhibitor of TGF-*β*, has also been administered in the experimental and clinical studies of diabetic nephropathy. It is known that CCN2 interacts with other growth factors involved in diabetes complications such as TGF-*β*1. CCN2 is one of the TGF-*β*1-inducible immediate early genes and acts downstream of TGF-*β*1 [85], participating in TGF-*β*1-induced cell proliferation and ECM synthesis. CCN2 exerts its fibrogenic effects through the TGF-*β*RII in both human proximal tubule cells (PTCs) and cortical fibroblasts (CFs) [86]. Based on the interaction of TGF-*β* and CCN2, anti-TGF-*β* strategies have been carried out in DN. In experimental model of diabetic nephropathy, tranilast was administered and demonstrated to attenuate tubulointerstitial pathology and albuminuria [87]. In PTCs and CFs, tranilast not only decreased TGF-*β*1-induced CCN2 mRNA and phosphorylation of Smad2, but also inhibited CCN2-induced ECM protein and cellular hypertrophy [88]. Furthermore, a small pilot clinical study demonstrated that tranilast decreased the decline in glomerular filtration rate in patients with advanced diabetic nephropathy over a 12-month period [89]. All these findings suggest that tranilast is a very promising renal protective compound partly through inhibition of CCN2 in DN.

## 4. Conclusions

Multifactorial pathogenic factors and mechanisms work together and lead to the development and progression of diabetic nephropathy (DN). At present, treatment strategies against DN are mainly focusing on better controlling of blood glucose, blood pressure, and blood lipids, as well as reaching the standard. Even though the patients with diabetic nephropathy have obtained several benefits from the current treatments, the long-term prognosis remains not satisfactory. Consequently, seeking for new treatment targets or therapeutic agents against diabetic nephropathy is urgently needed.

CCN2, as a profibrotic mediator and a proinflammatory cytokine in the development and progress of DN, has been payed more attention by nephrologists in the past decades. Some specific and nonspecific CCN2 inhibitors addressed in this review have shown potential renoprotective effects and highlight the future of the treatment of diabetic nephropathy. However, we should also realize that different modules in the CCN2 protein serve different functions and their functions are cell types specific in some extent [90, 91]. In addition, CCN2 mRNA stability is markedly influenced by its polyA tail, which has a pivotal effect on the posttranscriptional regulation of CTGF/CCN2 [92]. These findings enlighten us that treatment strategies targeting on CCN2 protein module or CTGF RNA stability in DN may be promising, and it needs to be undertaken and further investigated in DN. We anticipate that such studies could become reality in human disease and improve the prognosis of DN in the near future.

## Figures and Tables

**Table 1:** Agents for specific inhibition of CCN2 expression in diabetic nephropathy.

Agents	Subjects	Treatment plan	Pathway	Outcomes
CTGF ASO [27]	T1DM mice	20 mg/kg/2 qw for 16 weeks, subcutaneously	Inhibition of phosphorylation of p38 MAPK and CREB pathway	CCN2 ASO reduced CCN2 expression in the kidney of diabetic mice. CCN2 ASO decreased proteinuria and albuminuria.
	T2DM db/db mice	5, 10, and 20 mg/kg/2 qw for 8 weeks, subcutaneously		CCN2 ASO reduced serum creatinine and attenuated urinary albuminuria and proteinuria in diabetic mice.

FG-3019 [32]	T1DM, T2DM patients	3 or 10 mg/kg/2 qw, i.v., 8 wks		FG-3019 decreased urinary albumin/creatinine ratio (ACR).

T1DM: type 1 diabetes mellitus; T2DM: type 2 diabetes mellitus; IG: intragastric; HK2: human renal proximal tubular epithelial cells.

**Table 2:** Agents for nonspecific inhibition of CCN2 expression in diabetic nephropathy.

Agents	Subjects	Treatment plan	Pathway	Outcome
Losartan [43]	T1 DN patients	50, 100, and 150 mg/day for 2 months, then 100 mg for 36 months		Losartan persistently decreased urinary CCN2 excretion, which correlated with a slower rate of decline in GFR

Spironolactone [46]	MCs, PTCs T2DM rats	100 nM for 24 h; 20 mg/kg/day, p.o. for 8 months	TGF-beta1-independent pathway	Spironolactone suppressed the production of CCN2 in MCs, PTCs, and T2DM rat model. Spironolactone reduced urinary protein and albumin excretion.

Fasudil [52]	T1DM rats	10 mg/kg/day IG for 30 days	Rho/Rho-kinase pathway	Fasudil inhibited CCN2 expression in the renal cortex of diabetic rats, with no affection of plasma glucose, blood pressure, and creatinine clearance in the diabetic rats. Fasudil suppressed urinary excretion of albumin.

Fasudil [54]	HMCs	HG 30 mmol/L fasudil 25, 50, and 100 *µ*mol/L for 12, 24, 36, 48, and 72 h	Rho/Rho-kinase pathway	Fasudil reduced CCN2 mRNA expression and protein secretion.

Fluorofenidone [65] (AKF-PD)	MMC	TGF-*β*1 (1 ng/mL) fluorofenidone (2 mM) for 24 hours	ERK and p38 pathways	Fluorofenidone reduced TGF-*β*1-induced CCN2 expression.

Fluorofenidone [66] (AKF-PD)	HK2	TGF-*β*1 (5 ng/mL) AKF-PD 1 mM, 2 mM For 48 h	Downregulation of p-Smad2 and p-Smad3 proteins.	AKF-PD downregulated TGF-*β*1-induced CCN2 expression and attenuated EMT.

Exendin-4 [73]	HMC	HG 30 mmol/L Ex-4 0.03, 0.3, and 3 nmol/L for 24 hours	cAMP/PKA pathway	Exendin-4 decreased HG-induced the expression of TGF-*β*1 and CCN2.

PCB [78] PCE [78]	HRMC db/db mice	HG (33 mM) PCB 10, 20 *μ*g/mL for 3 days 10 mg/kg PCE, p.o. daily for 8 weeks.	IL-8-Tyk2-STAT signaling	PCB suppressed IL-8-instigated CCN2 expression and collagen IV deposition. PCE extenuated the expression of TGF-*β*, CCN2, and collagen IV in renal tissues, alleviated glomerulosclerosis and renal filtration dysfunction, and lessened heavy proteinuria.

PCA [79]	HRMC	HG (33 mM) PCB 10, 20, and 25 *μ*g/mL for 3 days	TGF-*β*-SMAD signal NF-*κ*B signaling MCP signaling	PCA attenuated HG-induced CCN2 expression and collagen IV production. PCA reversed HG-reduced MT-1 MMP and HG-augmented TIMP-2 expression. PCA inhibited HG-induced ICAM-1 and MCP-1 expression. PCA attenuated renal fibrosis and mesangial inflammation.

T1 DN: type 1 diabetes nephropathy; GFR: glomerular filtration rate; MCs: mesangial cells; PTCs: proximal tubular cells; T2DM: type 2 diabetes mellitus; IG: intragastric; HMCs: human mesangial cells; MMC: mouse mesangial cells; HK2: human renal proximal tubular epithelial cells; HRMC: human renal mesangial cells; PCB: purple corn butanol fraction; PCE: extracts of purple corn; PCA: purple corn anthocyanins; MCP-1: monocyte chemoattractant protein-1; ICAM-1: intracellular cell adhesion molecule-1.
